# Editorial: Cardiovascular Mechanobiology

**DOI:** 10.3389/fphys.2021.833941

**Published:** 2022-01-20

**Authors:** Markus Hecker, Dirk J. Duncker

**Affiliations:** ^1^Department of Cardiovascular Physiology, Heidelberg University, Heidelberg, Germany; ^2^Department of Cardiology, Erasmus University Medical Center, Thoraxcenter, Rotterdam, Netherlands

**Keywords:** mechanobiology, cardiac, vascular, wall tension, wall shear stress, cardiovascular disease

Biomechanical forces play a major role in organ development, shape, and function. When exceeding the physiological range, however, they can become detrimental for organ structure and function. This is probably best exemplified by the cardiovascular system, with both the heart and blood vessels being continuously exposed to the biomechanical forces exerted by the pressure and flow of blood, which produce not only acute changes in cardiovascular function but also result in structural changes in the cardiovascular system.

The field of cardiovascular mechanobiology is a fascinating and clinically highly relevant Research Topic, with many research groups contributing to the advancement of our understanding of the acute and chronic adaptations and maladaptations produced by biomechanical forces. These insights continue to generate novel targets for therapy of a variety of cardiovascular diseases. Several aspects of cardiovascular mechanobiology are presented in this very timely Frontiers Research Topic “*Cardiovascular Mechanobiology*,” containing a total of 10 articles, with a balanced mix of 5 review and 5 original articles.

In the first review article, Jackson discusses the mechanobiology of myogenic tone in peripheral resistance arteries and arterioles, presenting the current understanding of the multiple mechanisms that have been implicated in myogenic tone, including the roles played by G-protein coupled receptors, ion channels, and protein kinases in vascular smooth muscle cells. The article highlights the areas where our knowledge and understanding of myogenic tone has remained incomplete and that warrant further research.

In the second review paper, Sporkova et al. review the mechanisms underlying the mechanobiological cues mediating the inward remodelling of small arteries and arterioles in response to chronic stretch produced by, for example, hypertension. Particular focus is on the role that LIM domain containing proteins play in mechanosensing and transduction in vascular smooth muscle and endothelial cells, translating extra- and intracellular mechanobiological cues into changes in gene expression in these cells that drive vascular remodelling.

Brandt et al. review the mechanobiological aspects of microvascular function and structure in health and disease with a particular focus on the coronary microcirculation. An in-depth review is provided on the factors involved in coronary microvascular functional and structural alterations in obstructive and non-obstructive coronary artery disease and the molecular mechanisms involved, providing novel potential targets for the treatment of ischemic heart disease.

The mechanobiology of endothelial-to-mesenchymal transition and its role in cardiovascular disease is reviewed by Islam et al. highlighting the emerging role of shear stress, cyclic strain, matrix stiffness, and composition in endothelial-to-mesenchymal transition, which is a hot topic particularly in maladaptive cardiac remodelling due to pressure overload but also in response to myocardial infarction. The authors also identify areas requiring further investigation.

Finally, Lityagina and Dobreva review novel insight into the epigenetic aspects of cardiovascular mechanobiology. Specifically, they describe how mechanical forces are transduced to chromatin through the tensed actomyosin cytoskeleton, the linker of nucleoskeleton and cytoskeleton (LINC) complex and the nuclear lamina, and the importance of these mechanisms in cardiovascular disease.

In the original research articles various aspects of the mechanobiology of vascular smooth muscle cells, endothelial cells, as well as cardiomyocytes were investigated. Using inducible pluripotent stem cell (iPSC)-derived cardiomyocytes, Körner et al. tested the hypothesis that the environmental stiffness influences structural and functional properties of iPSC-derived cardiomyocytes. The authors observed that soft surfaces with stiffnesses in the physiological range improve the expression pattern and interaction of cardiac proteins relevant for excitation-contraction coupling. Moreover, soft substrates influenced contractile properties and improved intercellular coupling in iPSC-derived cardiomyocytes, indicating that mechanical stiffness of the cell environment drives iPSC-derived cardiomyocytes toward further maturation by inducing adaptive responses.

In the next article, Akinbote et al. investigated vessel-stroma crosstalk in normal conditions and in the presence of fibrosis, using human iPSC-derived endothelial cells co-cultured in the absence and presence of primary human cardiac and lung fibroblasts in a microfluidic device to generate cardiac and pulmonary-like microvasculature that can be perfused at near physiological flow rates. Their study not only demonstrates the strong impact of stromal-endothelial cell interactions on vessel formation and extravascular matrix regulation, but their human 3D *in vitro* set-up could be very useful in future studies examining anti-fibrotic therapies on patient-specific iPSCs.

Wall shear stress has been proposed to influence intracranial aneurysmal growth and rupture. Consequently, Morel et al. investigated the effects of low and supra-high aneurysmal wall shear stress on porcine arterial endothelial cells. The authors observed that differential regulation of gene expression observed under various wall shear stress conditions translates into a different organisation of the endothelial cell architecture, suggesting that this adaptation of endothelial cells to different aneurysmal wall shear stress conditions may affect vascular remodelling in intracranial aneurysms.

Myocardin related transcription factors (MRTFs), including myocardin itself, MRTF-A, and MRTF-B are co-factors of serum response factor (SRF) that activate the smooth muscle cell gene program and play a key role in smooth muscle cell differentiation and mechanobiology. Liu, Bankell et al. investigated the role of alterations in MRTF signalling in vascular inflammation, and observed a cell type specific (vascular smooth muscle but not endothelial cells) suppression of inflammatory mediators. In a second study, Liu, Rippe et al. studied the regulation of muscarinic receptors by MRTFs and observed that muscarinic receptor M3 expression was driven by MRTFs in a cell specific manner, i.e., by MRTF-B/SRF in endothelial cells and by myocardin/SRF in vascular smooth muscle cells.

These highly diverse review and original research articles, covering a wide range of studies, illustrate the broad biological implications of mechanosensing and mechanotransduction in the cardiovascular system. We wish you an interesting and enjoyable journey through this Frontiers Research Topic!

## Author Contributions

All authors contributed equally to the writing of this article and organisation of the Research Topic.

## Conflict of Interest

The authors declare that the research was conducted in the absence of any commercial or financial relationships that could be construed as a potential conflict of interest.

## Publisher's Note

All claims expressed in this article are solely those of the authors and do not necessarily represent those of their affiliated organizations, or those of the publisher, the editors and the reviewers. Any product that may be evaluated in this article, or claim that may be made by its manufacturer, is not guaranteed or endorsed by the publisher.

